# Detection and characterization of resting state functional networks in squirrel monkey brain

**DOI:** 10.1093/texcom/tgad018

**Published:** 2023-09-02

**Authors:** Anirban Sengupta, Feng Wang, Arabinda Mishra, Jamie L Reed, Li Min Chen, John C Gore

**Affiliations:** Vanderbilt University Institute of Imaging Science, Nashville, Vanderbilt University Medical Center, Nashville, TN, United States of America; Radiology and Radiological Sciences, Vanderbilt University Medical Center, Nashville, TN, United States of America; Vanderbilt University Institute of Imaging Science, Nashville, Vanderbilt University Medical Center, Nashville, TN, United States of America; Radiology and Radiological Sciences, Vanderbilt University Medical Center, Nashville, TN, United States of America; Vanderbilt University Institute of Imaging Science, Nashville, Vanderbilt University Medical Center, Nashville, TN, United States of America; Radiology and Radiological Sciences, Vanderbilt University Medical Center, Nashville, TN, United States of America; Vanderbilt University Institute of Imaging Science, Nashville, Vanderbilt University Medical Center, Nashville, TN, United States of America; Radiology and Radiological Sciences, Vanderbilt University Medical Center, Nashville, TN, United States of America; Department of Psychology, Vanderbilt University, Nashville, TN, United States of America; Vanderbilt University Institute of Imaging Science, Nashville, Vanderbilt University Medical Center, Nashville, TN, United States of America; Radiology and Radiological Sciences, Vanderbilt University Medical Center, Nashville, TN, United States of America; Biomedical Engineering, Vanderbilt University, Nashville, TN, United States of America; Vanderbilt University Institute of Imaging Science, Nashville, Vanderbilt University Medical Center, Nashville, TN, United States of America; Radiology and Radiological Sciences, Vanderbilt University Medical Center, Nashville, TN, United States of America; Biomedical Engineering, Vanderbilt University, Nashville, TN, United States of America; Department of Physics and Astronomy, Vanderbilt University, Nashville, TN, United States of America

**Keywords:** brain, BOLD fMRI, independent component analysis, resting state networks, squirrel monkey

## Abstract

Resting-state fMRI based on analyzing BOLD signals is widely used to derive functional networks in the brain and how they alter during disease or injury conditions. Resting-state networks can also be used to study brain functional connectomes across species, which provides insights into brain evolution. The squirrel monkey (SM) is a non-human primate (NHP) that is widely used as a preclinical model for experimental manipulations to understand the organization and functioning of the brain. We derived resting-state networks from the whole brain of anesthetized SMs using Independent Component Analysis of BOLD acquisitions. We detected 15 anatomically constrained resting-state networks localized in the cortical and subcortical regions as well as in the white-matter. Networks encompassing visual, somatosensory, executive control, sensorimotor, salience and default mode regions, and subcortical networks including the Hippocampus-Amygdala, thalamus, basal-ganglia and brainstem region correspond well with previously detected networks in humans and NHPs. The connectivity pattern between the networks also agrees well with previously reported seed-based resting-state connectivity of SM brain. This study demonstrates that SMs share remarkable homologous network organization with humans and other NHPs, thereby providing strong support for their suitability as a translational animal model for research and additional insight into brain evolution across species.

## Introduction

Blood oxygenation-level dependent (BOLD) MRI signals provide spatial and temporal information on brain functions and were first detected in rodent brain, followed by human subjects and macaque monkeys (Ogawa, Lee, Kay, et al. 1990; Ogawa, Lee, Nayak, et al. 1990; Kwong et al. 1992; Dubowitz et al. 1998). Spontaneous fluctuations of BOLD signals at low frequency (0.01–0.1 Hz) were first observed in the motor cortex of human brains (Biswal et al. 1995) and subsequently have also been detected in animals (Vincent et al. 2007; Kannurpatti et al. 2008; Pawela et al. 2008; Shmuel and Leopold 2008; Hutchison et al. 2010). Since then many regions exhibiting temporally correlated BOLD fluctuations in a resting state have been identified, and resting state functional Magnetic Resonance Imaging (rs-fMRI) has been widely used to detect intrinsic functional networks within the brain (Damoiseaux et al. 2006; van den Heuvel and Hulshoff Pol 2010; Smith et al. 2013). Using data-driven exploratory analyses of fMRI data, distinct resting state networks (RSNs) have been delineated which have been implicated in auditory, visual, somatosensory, sensorimotor, default-mode as well as executive control tasks and are often found to occur bilaterally within the human brain (Beckmann et al. 2005; Jafri et al. 2008; Smith et al. 2009). These findings have been found to be grossly consistent across subjects (Smith et al. 2009, 2013) making them a characteristic feature of human brain functioning. Moreover, resting state functional connectivity (rsFC) have been widely used to quantify changes between these functional circuits under different diseased conditions in humans such as in Alzheimer’s disease (Lustig et al. 2003), autism (Kennedy et al. 2006), depression (Anand et al. 2005), multiple sclerosis (Lowe et al. 2002), and more recently, drug addiction (Menon 2011; Sutherland et al. 2012; Lerman et al. 2014).

Animal models offer the opportunity to perform experimental manipulations to mimic neuro-disorders and to better understand the functional and structural organizations of the brain (O’Reilly et al. 2013; Wu, Yang, et al. 2017; Sengupta et al. 2022). Beyond the motivation to be used as a preclinical model for translational neuroscience research, interest in understanding the mammalian brain organization and its evolution motivates researchers to explore the functional architecture of different animal species especially non-human primates (NHPs) as they have gross neuroanatomies that are homologous to that of humans but also differ from them to varying degrees (Kaas 2012, 2020). Over the course of evolution, brain regions can duplicate, fuse, reorganize, or expand, changing the proportions of different regions as well as its microstructure and connectivity to support specific functions (Sereno and Tootell 2005; Hill et al. 2010). Detecting which features are conserved across species and which are different is key to understanding the biological basis of different behaviors. RS-fMRI is well suited for the detection of brain networks across species and evaluation of homologies between them. Recent work has suggested that although basic RSNs span all mammalian species, new RSN topology develops as brain complexity increases in higher animals (Hutchison and Everling 2012).

Here we report the detection of functional networks in the brains of squirrel monkeys (*Saimiri sciureus*), a species of considerable interest within the hierarchy of non-human primates. The squirrel monkey (SM) is of New World origins and diverged from the human lineage 10 million years before the macaque monkey, an Old World species, and so evolved along separate branches (Friedrich et al. 2021; Royo et al. 2021). Although genetic data can identify which genes are preserved across species, there is considerable interest in identifying anatomic and functional differences in brains, in particular between SM and other species of NHP.

Resting state functional circuits can be delineated using hypothesis-driven approaches in which seed locations are identified based on existing knowledge of regions which are engaged in functional responses (Joel et al. 2011). Correlation analyses of BOLD signals from two seed regions are then used to derive functional connectivity between them (Biswal et al. 1995; Lowe et al. 1998; Xiong et al. 1999). However, when the location of functional subunits is unclear, as is the case while studying a new species’ brain, data driven approaches such as Independent Component Analysis (ICA) may be used to detect functional circuits (Calhoun et al. 2001; Sengupta et al. 2021). ICA has the advantage of not requiring prior hypotheses about the location of functions within any given structure and hence is suited for identifying spatially independent coherent BOLD fluctuations within the brain. It has been previously reported that resting state networks detected using ICA matches traditional seed-based approaches (Beckmann et al. 2005) which also provides evidence of the robustness of these circuits.

The goal of this study is to delineate the intrinsic functional organization of the SM brain at a high magnetic field of 9.4T using ICA and compare it with human and related NHPs belonging to both New-world and Old-world species. Another goal was to characterize the functional connectivity between ICA components which can serve as foundation for future resting state research aimed to understand changes in these circuits under various conditions. This is the first study per our knowledge to detect resting state functional circuits from SM whole brain at high temporal and spatial resolution. The location of the circuits detected compares well with previously delineated circuits from humans and other NHPs, and the connectivities between them were found to corroborate with previous seed-based studies from SM brain.

## Materials and methods

### Animal preparation

A total of 16 young adult SMs (male = 13) were included in the study with a mean age of 2.76 ± 0.63 years during the MRI scan. During each MRI scan, the monkeys were anesthetized with isoflurane (0.8–1.2%) delivered in a 30:70 O_2_:N_2_O mixture, and mechanically ventilated with head and body stabilized in a magnetic resonance (MR)-compatible frame. Vital signals (cardiac and respiratory cycles, core body temperature, end tidal CO_2_, peripheral capillary oxygen saturation SpO_2_ via pulse oximetry) were monitored and maintained within appropriate ranges throughout each imaging session. The respiration pattern during each fMRI scan was recorded and later used as a regressor in data processing. The procedures were performed under an animal protocol approved by the Institutional Animal Care and Use Committee at Vanderbilt University. NIH and federal guidelines on the ethical use and care of research animals were followed.

### MRI data acquisition

All MRI scans were performed on a 9.4T Varian/Agilent MRI scanner using a quadrature birdcage volume coil (inner diameter 85 mm). High-resolution T_2_*-weighted anatomical images were acquired on 24 contiguous axial slices covering whole brain (TR (repetition time)/TE (echo time): 480/10 ms,0.125 × 0.125 mm^2^ in-plane resolution, 512 × 512 matrix, slice thickness 1 mm). FMRI data were collected for the same slices using a two-shot gradient echo planar imaging (GE-EPI) sequence (repetition time TR = 1,500 ms (2 shots), echo time TE = 16 ms, resolution 1 × 1 × 1 mm^3^, 3 s per volume, interleaved slices). An extra navigator echo was collected with no phase encoding before the acquisition of the actual image data. This echo is used to correct for phase variations typically caused by motion. In each MRI session, multiple rsfMRI runs (300/210 volumes per run, 2–4 runs each session) were acquired prior to acquiring stimulation runs (150 volumes per run, 3–6 runs each session). Note that for those resting runs which had 300-volume data, we considered the first 210 volumes for our analysis, thus ensuring uniformity across subjects in the study. Each resting session took 31.5 min considering 210 volumes with 3 runs each session, while stimulus runs took 45 min considering 6 runs each session. Ventilation rate was adjusted to match the TR of fMRI data acquisition.

### FMRI data pre-processing and analysis

The rsfMRI data underwent slice-by-slice, 2D motion correction implemented in MATLAB R2020b. Functional image volumes were first aligned using a 2D rigid body motion correction algorithm based on maximization of mutual information, by which three motion parameters were estimated (two translations and one rotation) (Mishra et al. 2019). Motion parameters (two translation and one rotation), along with temporal signals extracted from muscle and cerebral-spinal fluid (CSF) regions containing at least 70% of the cumulative variance (using principal components analysis) were considered as nuisance parameters and were regressed out using a general linear model (Chen et al. 2015; Barry et al. 2017, 2018; Wu et al. 2019). No spatial smoothing was performed. The fMRI signals were then corrected for physiological noise (respiratory and cardiac signal) using RETROICOR (Glover et al. 2000). The axial fMRI images were up sampled from 1 mm^2^ to 0.125 mm^2^ to match the anatomic images. The rsfMRI signals were band-pass filtered (Chebyshev type2 IIR filter, cut-off frequencies 0.01 and 0.1 Hz) prior to functional connectivity analyses. A temporal signal-to-noise mask (tSNR < 10) was used to eliminate non-brain voxels from the analyses. Occasionally, a manually outlined muscle mask was also used to eliminate muscle in the analysis. Any functional runs with motion parameters >1 mm and temporal SNR <16 was excluded from the analysis. We also analyzed the tSNR values from different brain regions to check for any systematic pattern that can influence the rest of the analysis (Supplementary Material).

For group level analysis we used the SM brain atlas (VALIDATE Atlas) (Schilling et al. 2017) as the template for registration. Initially, median fMRI images were co-registered to the structural T2*W image in an individual subject space using FSL affine registration (*flirt*) and the transformation was then applied to the fMRI time series data. In the next step each subject’s anatomic images were co-registered to the VALIDATE atlas using FSL non-rigid registration (*fnirt*) and subsequently the transformation was applied to the functional fMRI time series to perform the group analysis (Jenkinson and Smith 2001).

### Independent component analysis (ICA) of resting state fMRI data

The coregistered 2D resting state functional data from 14 monkeys having 37 runs were temporally concatenated, and group ICA was performed on the rsfMRI signals from the entire brain. Runs that qualified for the analysis varied per animal as follows: 2 runs (8 animals), 3 runs (3 animals), and 4 runs (3 animals). Standard procedures in GIFT software (Calhoun et al. 2001) were followed to obtain spatial ICA maps and their corresponding time series. Next, we used a dual regression technique to obtain subject-specific component maps, along with their associated time series (Beckmann et al. 2009). In spatial ICA, each component generally denotes a network which is spatially independent from other networks. For ICA decomposition of the entire brain, we chose 15 components based on previous reports of studying the brain of other species using ICA (Beckmann et al. 2005; Damoiseaux et al. 2006; Smith et al. 2009; Hutchison et al. 2011; Belcher et al. 2013; Hori, Schaeffer, Gilbert, et al. 2020) and empirical evidence of synchronized BOLD fluctuations in specific regions of the SM brain using different range of components such as 10, 15 and 20 (more details in Supplementary Material).

The reliability of the estimated group ICs was obtained using GIFT software’s ICASSO method (Himberg et al. 2004). The algorithmic and statistical stabilities were investigated by running the algorithm 10 times with different initial values or/and with differently bootstrapped data sets, respectively. We also performed single subject ICA from individual monkeys with parameters matched to group ICA data to examine the robustness of the group ICs at an individual subject level.

### IC spatial location and resting state network identification

To identify the location of the ICs, we used one digital SM atlas (Schilling et al. 2017) and two stereotaxic atlas (Gergen and MacLean 1962; Akert 1963). The spatial component maps were thresholded (z-score > 3) and were identified to belong to different cortical gray matter (GM) and white matter (WM) regions by overlapping them with the digital atlas which had different GM and WM regions classified. The subcortical regions were identified based on visual inspection and comparing them with the two stereotaxic SM brain atlas. The classification of the components in terms of RSNs was performed by comparison with known functional networks previously identified from ICA studies in humans (Beckmann et al. 2005; Damoiseaux et al. 2006; Smith et al. 2009), macaques (Hutchison et al. 2011; Yacoub et al. 2020) and marmosets (Belcher et al. 2013).

### Functional connectivity measure

The spatial location of each component obtained from ICA, often found bilaterally in the brain, was considered as the region of interest (ROI) for further evaluation. Functional connectivity between all such ROIs (derived from the group ICA of 14 monkeys) was computed at the individual subject level using the mean fMRI time series of each ROI for the initially recruited 14 monkeys plus 2 additional. The rationale is once the ROIs are standardized from a sufficient sample size (14 monkeys in our study), those ROIs can be used for other monkeys of the same species (more details in Supplementary Material). Connectivity was measured as the Pearson’s correlation coefficient (r value) between the mean time courses of pairs of ROIs and represented as a connectivity matrix. The elements (r value) in the matrix for which p < 0.05 (False-Discovery Rate or FDR corrected) were considered as statistically significant. Finally individual subject connectivity matrixes were averaged from 16 monkeys (43 runs) to obtain the mean connectivity matrix and elements of this matrix which were considered significant if their median value over the individual subjects was significant after FDR correction.

### Network analysis using graph theory

A simple network analysis was performed on the connectivity matrix using the Gephi software (Bastian et al. 2009) based on graph theory principles (Rubinov and Sporns 2010). In graph theory, a network is defined by a collection of nodes (vertices), and links (edges) between pairs of nodes. Nodes in our study represent the ROIs (from all the slices), while links represent functional connectivity values between those ROIs. We also computed the node strengths, which are the sum of weights of links connected to each node, averaged over the number of links to the node.

### Fractional amplitude of low-frequency fluctuations (fALFF)

Several methods have been developed to examine the amplitudes of low-frequency oscillations (Wang et al. 2014). Amplitude of low-frequency fluctuations (ALFF) has been considered a useful tool to depict local brain activity. ALFF reflects brain activity level during a period of time and has been used to detect brain activity abnormalities in a spectrum of brain diseases, such as mild cognitive impairment, attention deficit hyperactivity disorder and post-traumatic stress disorder (Yu-Feng et al. 2007; Han et al. 2011; Yin et al. 2011). In the current study, we measured the fractional amplitude of low-frequency fluctuations (fALFFs) as a measure of the strength of the low-frequency oscillations, which is the ratio of the power of low-frequency signals (0.01–0.08 Hz) to that of the higher frequency range (0.08–0.15 Hz). Power was calculated by computing area under the power spectrum within the frequency range.

## Results

### Independent component analysis of SM brain fMRI

**Fig. 1:**
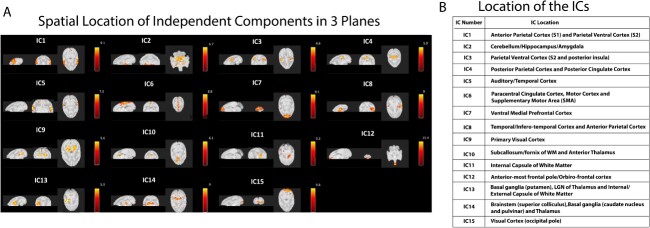
15 independent components detected using group ICA (N = 14). A) Spatial location of the 15 independent components in 3 orthogonal directions: Sagittal, coronal and axial from the most informative slice (thresholded at Z = 3). B) The location of the components is specified here.

15 independent components were extracted using ICA from the whole brain fMRI data of 14 SMs. Figure 1A shows the spatial location of these 15 components in sagittal, coronal and axial orientations, from the most representative axial brain slice. Figure 1B names the locations of these components after reference to the SM brain atlas. Supplementary Figure S1 shows the 15 independent components in axial orientation from all the slices of the whole brain.

**Detailed descriptions** of the locations of the 15 networks and their proposed functional roles are given below. Components were found bilaterally unless otherwise noted.

#### Cortical components

**IC1:** This component is located in the Anterior Parietal Cortex (APC) corresponding to the Primary Somatosensory cortex or S1 (also contains BA,1,2,3a and 3b) as well as in the Parietal Ventral Cortex (PVC) containing Secondary Somatosensory Cortex or S2.

**IC3:** This component is located in the APC along with areas from PVC/S2 including Posterior Insula (PIns). It also includes Anterior Cingulate Cortex (ACC) over the medial brain.

**IC4:** This component is located in the Posterior Parietal Cortex (PPC) and Posterior Cingulate Cortex (PCC) containing the sensorimotor regions. It has a medial appearance from middle to inferior brain slices.

**IC5:** This component is located in the Auditory/Temporal Cortex and has a lateralized appearance in the middle to inferior brain slices.

**IC6:** This component encompasses the Paracentral Cingulate Cortex network located medially in the superior brain slices. It Includes medial wall, ACC, PCC and also has overlap with the motor cortex (M1) and supplementary motor area (SMA).

**IC7:** This component is located at ventral medial Prefrontal Cortex (vmPC) including the frontal pole and is located at the anterior most part of the middle to inferior brain slices.

**IC8:** This component encompasses parts of the infero-temporal Cortex and APC. It has a unilateral appearance from the middle to inferior brain slices.

**IC9:** This component has a medial appearance in the Primary Visual Cortex and represents the higher order midline visual network of the brain.

**IC12:** This component is located in the anterior-most frontal pole region overlapping with the Orbito-Frontal Cortex (OFC) at the inferior slices of the brain.

**IC15:** This component is located medially in the occipital pole of the Visual Cortex region.

#### Sub-cortical and white matter components

**IC2:** This component encompasses the Hippocampus-Amygdala region and some overlap with the cerebellum. It is located in inferior slices toward the mid-anterior portion of the brain.

**IC10:** This component is located in the Subcallosum/fornix of WM with limited overlap over the anterior thalamus in the mid-inferior slices of the brain.

**IC11:** This component is located unilaterally in the Internal Capsule (WM) region in the mid-inferior slices of the brain.

**IC13:** This component contains basal-ganglia structures such as putamen, lateral geniculate nucleus (LGN) of the thalamus and overlaps with the internal and external capsule of WM in the mid-inferior slices of the brain.

**IC14:** This component is primarily observed in the superior colliculus of brainstem and contains parts of the basal-ganglia structures such caudate nucleus and pulvinar as well as parts of the thalamus. It is located medially in the mid-inferior slices of the brain.

### Robustness of independent components

We also tested the reliability of the estimated ICs using GIFT software’s ICASSO method (Himberg et al. 2004). The reliability of the ICs was found to be high with a stability index greater than 0.97 for all the components indicating they are concentrated in compact and close-to-orthogonal clusters and highly consistent across multiple ICA iterations. The components accounted for 71.79% of the data’s variance. We also tested for the spatial robustness of the components with 2 new monkeys (6 runs) added to the initial cohort of 14 monkeys. What we found is a gross similarity between the ROIs obtained before using 14 animals (37 runs) and those obtained using the 16 animals (43 runs) at the same threshold, with all the 15 components being detected in the new cohort. The details of the results are provided in Supplementary Fig. S2.

**Fig. 2:**
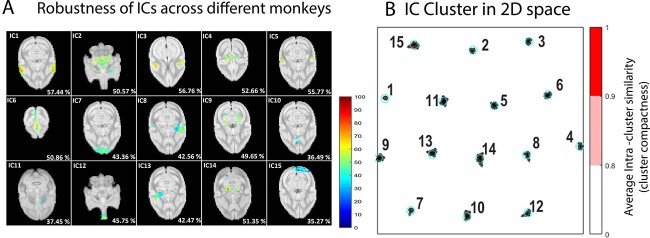
Reliability results of independent components. A) Percentage occurrence of the 15 independent components across the 14 monkeys (37 runs) from a representative slice. Voxels thresholded at a value of 1 at the group level ROIs were considered. The mean percentage from all the ROI voxels in that slice are provided at the bottom right of each component’s panel. All the components are overlayed on squirrel monkey brain (VALIDATE) atlas (Schilling et al. 2017) for display purpose. B) Intra-cluster similarity of the spatial location of the IC estimates through a projection on a 2D space. Compact and isolated clusters suggest reliable estimates. The distance between clusters represents the Euclidean distances between them in two dimensions. Note pair-wise similarity between estimates inside a cluster is omitted if the intra-cluster similarity is above 0.9.

To investigate the robustness of the group identified ICs across the individual monkeys, we analyzed the individual subject specific ICA maps obtained from the dual-regression technique. Figure 2A shows the percentage of occurrence of each of the 15 ICs from the 14 monkeys (37 runs) from a representative slice. The majority of the cortical components viz. somatosensory cortex (IC1), parietal cortex (IC3), cingulate cortex along with paracentral network (IC4 and IC6 ), temporal/auditory cortex (IC5) and the primary visual cortex (IC9) as well as the sub-cortical components viz. amygdala/hippocampus/cerebellum (IC2) and the brainstem/thalamus (IC14) regions were found to occur robustly (mean percentage > 50%) across all the monkeys. Components which were the less robust (mean percentage < 40%) was found to be from the WM (IC10 and IC11) and occipital pole (IC15). Figure 2B shows the 2D projection of the estimated clusters of the ICs over multiple iterations of ICA. All the estimated clusters showed high intra-cluster similarity (>0.9) which is also evident through their compact spatial profile.

### Connectivity between independent components

**Fig. 3:**
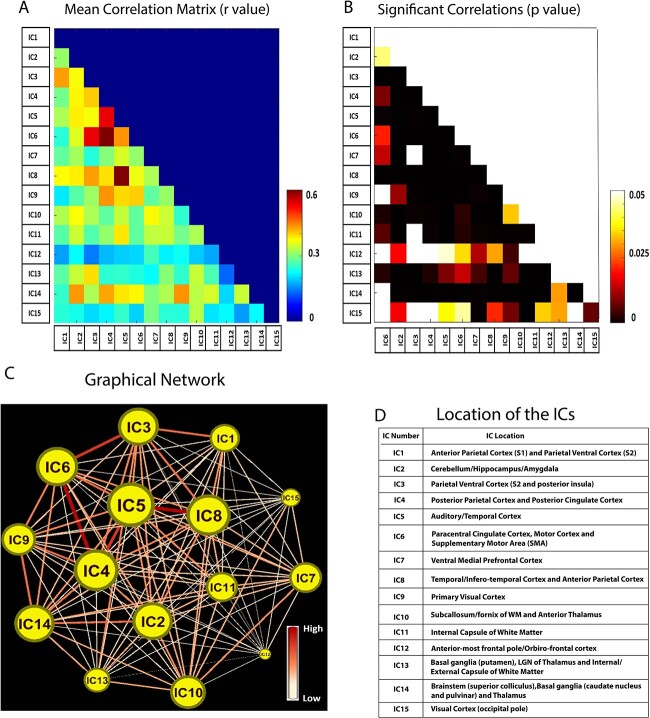
Correlation between independent components (N = 16). A) Mean correlation matrix (absolute r values) from the 15 ICs and B) FDR corrected significance (p) values for each of these correlations. All the non-white boxes from the lower triangle are significantly correlated (p < 0.05). C) Graphical representation of the connectivity between the different ICs whose locations are given in D). The nodes in the graph represent each IC and the edges represent the correlation between them. Nodes with higher node strength are bigger in size and edges with higher correlation values (0 < r < 1) appear darker and thicker.

Once the ROIs from the 15 independent components were standardized from the sample of 14 monkeys, we performed connectivity analysis from those 14, as well as 2 more monkeys which were added later. Thus, the ICA detected ROIs were examined for connectivity analysis from 43 runs of 16 animals. Runs that qualified for the analysis varied per animal as follows: 2 runs (8 animals), 3 runs (5 animals), and 4 runs (3 animals). Pairwise correlation between the time-course of different ICs were computed, averaged over 16 monkeys and represented as a connectivity matrix as shown in Fig. 3A.

Most of these connectivities were significantly correlated (FDR corrected p < 0.05) as revealed in Fig. 3B in which statistical p values are presented for each pair of connectivity. Figure 3C shows a graphical representation of the connectivity between these different networks whose locations are provided in Fig. 3D. The networks which had higher node strength had bigger size. The graph further shows that IC4 (PPC and PCC), IC5 (Temporal/Auditory Cortex) and IC8 (infero-temporal cortex and APC) located at the center of the graph had the highest node strength (node strength > 5) whereas networks IC12 (frontal pole of OFC) and IC15 (occipital pole of Visual cortex) had the lowest node strengths (node strength < 3.3). The connections which possessed the highest significant correlation (r > 0.5) were between IC3-IC6 (PVC/S2/PIns- Paracentral Cingulate Cortex), IC5-IC8 (Auditory-APC), and IC6-IC4 (Paracentral Cingulate Cortex-PPC/PCC) as shown by the thick red edges connecting these nodes. Other networks which were significantly correlated with a high r value (0.4 < r < 0.5) were IC1-IC3, IC4-IC3, IC5-IC6, IC3-IC8, IC4-IC9, IC6-IC9, IC2-IC14, and IC9-IC14 with semi thick orange edges connecting their nodes.

### Power spectrum analysis of the independent components

**Fig. 4:**
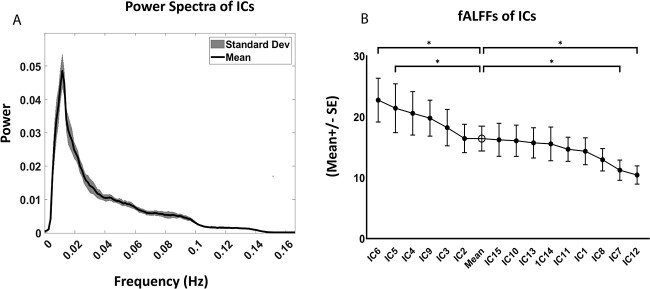
Frequency analysis of the independent components from the 14 monkeys. A) Averaged Power spectrum (Mean +/− SD) from the 15 ICs obtained from group analysis. B) Line plot of the Mean +/− Standard Error (SE) fALFF values (N = 14) of each IC arranged in a descending order. Significantly different values compared to the mean fALFF (from all the components) are denoted by * (p < 0.05) using one-way ANOVA technique. fALFF was calculated as the ratio of the power of low-frequency signals (0.01–0.08 Hz) to that of the higher frequency range (0.08–0.15 Hz) for each of these components.

The Mean ± SD of the power spectrum from all ICs is shown in Fig. 4A. The spectrum shows a typical increase in power at the lower frequency range (0.01–0.04 Hz) after which its power shows a gradual fall for all ICs. A line-plot of the Mean ± SE of the fALFF values of these components arranged in descending order is shown in Fig. 4B. Components such as IC4 (PPC, PCC), IC5 (Auditory), IC6 (Paracentral Cingulate cortex & SMA) and IC9 (Primary Visual Cortex) show mean values greater than 20 whereas certain other components such as IC7 (vmPC) and IC12 (OFC) had mean values of 11 or less. Performing a One-way ANOVA test revealed that IC5 and IC6 have significantly (p < 0.05) higher values than the mean fALFF obtained from all the components, while IC7 and IC12 had values significantly lower (p < 0.05).

## Discussion

Of the limited number of species examined with rs-fMRI, the rat and macaque, an old-world monkey, represent the largest proportion of animal investigations, partially owing to their widespread and ongoing contributions across multiple areas of neuroscience (Hutchison and Everling 2012). The evolution of primates is characterized by an increase in the surface of the cerebral cortex and expansion and subdivision of the neocortex (Lewis and Van Essen 2000; Kaas 2012; Friedrich et al. 2021). Although it is possible to study many facets of human brain functioning with rodent models, phylogenetic proximity to humans, larger pre-frontal cortex than rodents, similar brain architecture, patterns of neural activities in interconnected circuits and behavioral repertoire are among the most compelling reasons that nonhuman primates provide the ideal animal model for correlating with humans and also for testing the efficacy and the safety of treatments before entering clinical trials in humans (Perretta 2009).

Among non-human primate models, Old World monkeys (e.g. macaques etc.) and New World monkeys (e.g. squirrel monkeys and marmosets) are frequently used in cognitive and clinical neuroscience studies (Royo et al. 2021). However, from a practical perspective the use of macaques is made difficult by constraints such as their cost, handling procedures, housing requirements, and high gestation time. Also, work on macaques is costly and labor intensive leading to generally low sample size per experiment which is often too small to arrive at any generalized conclusion while comparing with humans. SMs on the other hand are easier to handle, have lower maintenance cost and shorter lifespan (~21 years in captivity compared to ~35 years for macaques) (Brady 2000). They also have considerably smaller body size (318 mm for males and 316 mm for females) and body weight (554 g–1,150 g for males and 651 g–1,250 g for females) (Rowe 1996; Bernacky et al. 2002) than macaques which makes them easier to breed and raise in captivity and also better matched to smaller bore size ultra-high field magnets. Common marmosets are another NHP new world species which has advantages of small size and ease in handling, but SMs have larger brains with more pronounced gyrification (Royo et al. 2021), and their performance on cognitive tasks are considered superior to marmosets (Williams and Glasgow 2000). These differences may be attributed to a higher volume ratio between the neocortex and the rest of the brain in SMs (69%) than in marmoset (60%) (Sawaguchi 1992). The cortex of SMs is also closer to the brain of macaques and very similar to humans except for some gyri and sulci that are also absent in the other monkeys (Royo et al. 2021).

SMs are increasingly being used in preclinical behavioral studies, pharmacological studies, electrophysiology and imaging studies (Brady 2000; Williams and Glasgow 2000; Chen et al. 2007; Wu, Wang, et al. 2017; Wu et al. 2019). The long enough life expectancy of SMs (~21 years in captivity) and their neurophysiological characteristics akin to humans offers an excellent opportunity for studying brain aging including Alzheimer’s (Fischer and Austad 2011), neurodegenerative diseases such as Parkinson’s (Purisai et al. 2005), and motor recovery mechanisms after stroke (Nudo et al. 2003; Barbay et al. 2006). Moreover, injury models (Chen et al. 2015; Sengupta et al. 2021) or age-related cerebral impairments (Elfenbein et al. 2007; Emborg 2007) are easy to replicate in SMs making it a very apt model for neurological clinical translational studies. Inspite of these advantages of SMs over others, to date there have not been comprehensive studies looking into whole brain resting state functional circuits of SM brain using fMRI.

This study reports the application of group ICA to anesthetized SM whole brain fMRI data acquired at sub-millimeter resolution using a 9.4T scanner. Using ICA, we obtained 15 anatomically constrained networks from SM brain that we postulate are engaged in different functions. These networks were highly robust across multiple iterations of ICA and many of them were consistently observed at the individual subject level. Most importantly these networks bear high correspondence with networks previously observed in humans (Beckmann et al. 2005; Damoiseaux et al. 2006; Smith et al. 2009) and those reported in other NHP species such as macaques (Hutchison et al. 2011) and marmosets (Belcher et al. 2013), as discussed below.

### Similarity of networks across species

#### Cortical networks

We postulate that IC9 and IC15 represent the **visual cortex** within SM brain with a medial location in the occipital lobe and at the occipital pole respectively. These components have been ubiquitously reported in human (Fig. 1A and E in Damoiseaux et al. 2006; Maps 1, 2, 3 in Fig. 1 of Smith et al. 2009), macaque (Fig. 1I in Hutchison et al. 2011), and marmoset (Fig. 2A, C, E and F in Belcher et al. 2013) literature. We did not observe a lateralized appearance of these components in the group level as is found in humans which can be because of the closed eye state during recording. The primary purpose of the visual cortex is to receive, segment, and integrate visual information (Huff et al. 2023). The processed information from the visual cortex is subsequently sent to other regions of the brain to be analyzed and used. The **somatosensory components** (IC1, IC3 and IC6) detected in our study over the PVC, APC and paracentral cingulate cortex region of SM brain have also been universally reported in humans (Fig. 1F in Damoiseaux et al. 2006), macaque (Fig. 1A in Hutchison et al. 2011) and marmoset (Fig. 2D and K in Belcher et al. 2013). Also, previous studies in SM brain from our group and others have identified S1 & S2 (IC1 & IC3) as regions of high BOLD activity in response to nociceptive stimuli and detected rsFC connectivity within the local circuits of these regions (Chen et al. 2007; Wang et al. 2013; Wilson et al. 2016; Wu, Wang, et al. 2017; Yang et al. 2018; Ye et al. 2021). While the primary function of S1 is to detect sensory information from the body regarding temperature, proprioception, touch, texture, and pain, S2 is associated with spatial and tactile memory associated with sensory experiences (Raju and Tadi 2023). The **sensorimotor component** (IC4) is another widely reported network in humans (Map 6 in Fig. 1 of Smith et al. 2009; Fig. 2 of (Raichle 2015)) and macaque (Fig. 1J in Hutchison et al. 2011) but was not detected in a similar position in marmosets. The major function of this network is sensorimotor integration which is the ability to incorporate sensory inputs that provide information about one’s body and the external environment to inform and shape motor outputs (Wolpert et al. 1998).

Another component was located at the Auditory/temporal cortex (IC5 & IC8) region of the brain which forms the **Auditory network**. This component is detected alone as well as in conjunction with other regions e.g. the occipitotemporal pathway and has been widely reported in humans (Fig. 1G and I in Damoiseaux et al. 2006; Maps 7 in Fig. 1 of Smith et al. 2009), macaques (Fig. 1A and D in Hutchison et al. 2011), and marmosets (Fig. 2H in Belcher et al. 2013). The auditory cortex is involved identifying the location of sound in space and its processing (Mtui et al. 2020). The temporal lobes are also believed to play an important role in processing emotions and certain aspects of visual perception (Mtui et al. 2020). The networks described above encompassing sensory and motor areas, including the visual, auditory, sensorimotor and somatosensory regions are generally considered to be of lower order in a cognitive processing hierarchy (Kaas 2012, 2020).

We also found parts of components IC4, IC6 and IC7 show significant correspondence to the previously described **Default Mode Network** (DMN) in humans (Fig. 1 in Damoiseaux et al. 2006; Maps 4 in Fig. 1 of Smith et al. 2009), macaques (Fig. 1C in Hutchison et al. 2011) and marmosets (Fig. 2G Belcher et al. 2013). The brain’s DMN consists of discrete, bilateral and symmetrical cortical areas, in the medial and lateral parietal, medial prefrontal, and medial and lateral temporal cortices of the human, nonhuman primate, cat, and rodent brains (Raichle 2015). However, there are slight differences between species as the authors of the macaque brain network (Hutchison et al. 2011) reported that there is absence of dorsal medial prefrontal cortex within the macaque DMN. The DMN network in SM brain as found in our study is divided into separate components including the PCC (IC4 and IC6) and the vmPC (IC7). Such partitioning of large-scale networks using ICA, including into dorsal and anterior portions of the DMN (Abou-Elseoud et al. 2010) has been reported in human and monkey fMRI literature before (Tian et al. 2013). The DMN has been found to be more active during passive tasks than tasks demanding focused external attention (Buckner 2013). One hypothesis behind this is that the DMN contributes to internal modes of cognition used when remembering, thinking about the future, and mind wandering.

The vmPC and frontal pole region (IC7 & IC12) also separately constitutes the **executive control network** which is known to provide bias signals to other areas of the brain in order to implement cognitive control (Friedman and Robbins 2022) and is frequently reported in humans (Fig. 1K and J in Damoiseaux et al. 2006; Maps 4 in Fig. 1 of Smith et al. 2009), macaques (Fig. 1E in Hutchison et al. 2011) and marmosets (Fig. 2I and L Belcher et al. 2013). Recent work proposes that dorsal ACC and insula constitute the brain’s **Salience network** (Seeley et al. 2007) and it features extensive connectivity with limbic and subcortical structures. IC3 in our study comprising the Pins and ACC along with other subcortical structures (described below) viz. IC2, IC13 and IC14 resembles the **Salience network** previously noted in humans and other animals (Fig. 2 in Raichle 2015), macaques (Fig. 1G in Hutchison et al. 2011) and marmosets (Fig. 2H in Belcher et al. 2013). The Salience network is critical for detecting behaviorally relevant stimuli and for coordinating the brain’s neural resources in response to these stimuli (Seeley et al. 2007). These networks involved in executive control, salience behavior and default-mode activity represent higher order processes in a cognitive processing hierarchy.

#### Subcortical networks

We also detected components located in the subcortical regions of the brain. One such network was located in amygdala and hippocampus region primarily, with little overlap of the cerebellum (IC2). The amygdala is specialized for input and processing of emotion, while the hippocampus is essential for declarative or episodic memory (Yang and Wang 2017). During emotional reactions, these two brain regions interact to translate the emotion into particular outcomes. Components were also detected in the basal-ganglia structures (IC13 and IC14) including putamen, caudate nucleus and pulvinar and at the LGN of thalamus (IC13). The basal-ganglia structures are responsible for different areas of the brain to work together (Lanciego et al. 2012) while the thalamus acts as the body’s information relay station where all information from the body’s senses (except smell) is processed before being sent to the brain’s cerebral cortex (Torrico and Munakomi 2023). Another IC was located at the superior colliculus of the brain stem region (IC14) which is an area where visual, auditory and somatosensory information are integrated to initiate motor commands (Ito and Feldheim 2018). These ICs (IC2, IC13 & IC14) represent *subcortical networks* of the brain which have been less robustly observed in other species although parts of it have been reported previously in humans (Cerebellum Map 5 in Fig. 1 of Smith et al. 2009, thalamus in Fig. 6F in Beckmann et al. 2005), macaque (hippocampus in Fig. 1K in Hutchison et al. 2011) and marmoset (basal-ganglia caudate and putamen Fig. 2B, parts of thalamus in Fig. 2H and cerebellum Fig. 2J in Belcher et al. 2013).

While the original marmoset study detected certain sub-cortical components (Belcher et al. 2013), more recently another group (Hori, Schaeffer, Yoshida, et al. 2020) detected fine sub cortical networks at hippocampus, caudate, putamen, Inferior Colliculus, LGN and various thalamic nuclei by following a fingerprinting approach in which the ICA obtained cortical networks were correlated with previously identified sub-cortical regions in the marmoset brain. This was motivated by the assumption that sub-cortical regions often suffer from lower spatial and temporal SNR, and as a result are not detected at rates similar to cortical networks while doing whole brain ICA. In our study we were able to detect most of these sub-cortical networks with whole brain data-driven ICA and we didn’t notice any systematic difference between the tSNR of the cortical and sub-cortical regions (Supplementary Fig. S3).

The authors from an earlier macaque brain study reported that components detected from their data were mostly restricted to cortical areas although whole brain coverage of macaques was ensured (Hutchison et al. 2011). However a recent study has detected sub-cortical networks located at parts of thalamus, hypothalamus, brainstem, amygdala, hippocampus and cerebellum region by using a high model order (40 independent components) which is known to reveal short range networks in the brain (Yacoub et al. 2020). Although contribution from broad sub-cortical regions was discussed in that study, finer hubs such as LGN, caudate or other thalamic nuclei, or superior or inferior colliculus of the brainstem region were not reported.

#### White matter networks

ICs were detected in the WM of SM brain (IC10, IC11 and IC13) which indicate coherent BOLD fluctuations within that region at resting state. Many WM tracts of the brain are known to be composed of myelinated axons (nerve fibers) that connect neurons from different functional brain regions thus providing structural connections between them (Mtui et al. 2020). IC10 in SM brain contains the Subcallosum/fornix WM bundles involved in connecting various subcortical structures. IC11 and IC13 contains internal and external capsule of WM fibers which are involved in communication between cerebral cortex and areas of the brainstem and other subcortical structures. Although various reasons (Gore et al. 2019) have precluded reports of WM connectivity in fMRI literature, our results provide evidence of the presence of WM correlations in BOLD fMRI signals of the brain and supports recent findings from WM literature (Ding et al. 2018; Gao et al. 2020; Li et al. 2020; Mishra et al. 2020). Hutchison et al. (2011) in their macaque brain study detected the presence of subcomponents II and III of the superior longitudinal fasciculus (WM) that connects posterior cortex to frontal cortex and inferior longitudinal fasciculus (WM) that connects the occipital to temporal cortex. Recently, spontaneous BOLD fluctuations were also detected in human brain using ICA (Huang et al. 2020). Note however that our method used a whole brain analysis in which the larger BOLD fluctuations in GM may dominate the smaller WM signals. However, in the human brain study (Huang et al. 2020) a separate WM mask was created and only signals from within the WM were analyzed so that the signal does not get dominated from that of GM.

### Robustness of the independent components

The group level ICs were highly robust as evident from their strong intra-cluster similarity (>0.9) across multiple iterations of ICA. The sample size of 14 monkeys was large enough to obtain the standardized ROIs, as adding new monkeys to the sample size didn’t alter the spatial location of the ICs with only mere difference in the IC decomposition process which is well known from ICA literature (Tian et al. 2013). The group level ICs that were most robust across the individual SM subjects were those from the cortical regions and the sub-cortical regions with a mean percentage of occurrence ≥ 50% over the 37 runs from all the voxels of the thresholded ROI. Note that the mean percentage of occurrence in the Fig. 2A is derived from those voxels that qualified the group level threshold of 1. Lowering this threshold will yield a higher percentage of occurrence across individual subjects. However, some of the components were less robust across the runs with a mean percentage of occurrence <40%, which could reflect individual differences in structural or functional connectivity within monkeys. Variability could also arise from the individual physiological state of each animal (due to anesthesia level or its age) or due to lower tSNR (Supplementary Fig. S3) at the peripheral brain regions where networks such as IC7, IC12 and IC15 were located. Overall, the cortical and sub-cortical networks were robustly obtained at the individual subject level of SMs while the WM networks (IC10 and IC11) were less robust (mean percentage < 40%) across them.

### Strong functional connectivity between cortical components corroborating previous seed-based results in SMs

In a previous study, the authors (Wu, Wang, et al. 2017) studied whole brain resting state connectivity of SM using seeds located at the two contiguous areas of S2 and PIns which were identified as core regions in nociceptive processing and pain perception. Spatial similarity and overlap analysis identified each region as part of two distinct intrinsic functional networks which have 7% overlap, one of which is the PIns-ACC-PC (Parietal cortex including APC and PPC)-PPC-Auditory networks. In our study, we find high correlation (r > 0.4) between IC6 (including ACC and PCC) and IC4 (including PPC and PCC) as well as between IC3 (PVC/S2/PIns and APC) and IC6/IC4. High correlation is also observed between IC5 (Auditory cortex) and IC4 and also between IC5-IC8 (infero-Auditory/temporal cortex and APC) all of which forms part of this network. The other network documented in that study was the S2-S1-area 7b-PC-ACC. In our study we observed high correlations between IC1-IC3 (S1/S2-PVC/S2) as well between IC3-IC8 (PVC/S2-APC) which matches well with the previously detected seed based resting state network (Wu, Wang, et al. 2017) thus corroborating our ICA findings with the previous seed-based report. The other major cortical connectivities (r > 0.4) detected in our study involved the primary visual cortex (IC9) which was found to have strong correlations with both cortical (IC4 and IC6) as well as sub cortical parts of the brain such as IC14 (brainstem and parts of the basal-ganglia structures).

### Strong connectivity of thalamus with cortical and sub cortical components

Thalamus is a key relay and integration center for sensory processing in the brain (Basso et al. 2005). The S1 receives neuronal projections from the thalamus and anatomical thalamo-cortical connections have been extensively studied in non-human primates (Davis et al. 1998; Huffman and Krubitzer 2001; Baxter 2013; Saalmann 2014; Pelekanos et al. 2020). The networks involving thalamus (IC13 and IC14) detected in our study were found to be strongly connected with both sub cortical networks such as IC14-IC2 (cerebellum-Hippocampus-Amygdala) and cortical networks involved in sensory processing such as IC13-IC3 (PVC/S2 and ACC), IC14-IC4 (PPC and PCC) and IC14-IC9 (primary visual cortex), thus confirming the role of thalamus in relaying motor and sensory information to the cerebral cortex.

### Differential fALFF values across the components

All the ICs followed a similar pattern of high power at low frequency range (0.01–0.04 Hz) followed by a gradual decrease in power in the higher frequencies (>0.04 Hz). However, a closer look revealed their difference in fALFF values. In general, most cortical components had higher than mean values (IC6, IC5, IC4, IC9 and IC3) with those from paracentral cingulate cortex/M1/SMA (IC6) and auditory cortex (IC5) being significantly higher than the mean. Components belonging to sub-cortical region (IC13 & IC14) as well as those encompassing the WM (IC10 and IC11) had lower fALFF than the mean but those from the vmPFC and OFC (IC7 and IC12) were significantly lower. Previous research in humans reported that low-frequency fluctuations are more detectable within GM than WM (Biswal et al. 1995; Zang et al. 2007; Zou et al. 2008; Zuo et al. 2010) which finds semblance in squirrel monkeys too from our analysis.

### Limitations

One limitation of using ICA for extracting resting state networks is that there are currently no well-established criteria to guide the selection of an optimal number of independent components for a given data set (Cole et al. 2010). Functionally connected regions can split into small subcomponents when the data are decomposed using higher numbers of components viz. 40 components were extracted for macaque brain by one research group (Yacoub et al. 2020), since larger numbers of components are needed to identify fine scale networks. Thus, there is a tradeoff between using higher model orders and more conservative lower model orders which are required to detect large scale networks. When we performed our analysis using 10 components, it helped avoiding separation of certain networks with multiple nodes viz. somatosensory and executive networks (Supplementary Fig. S4). However, it also led to less robust networks stay undetected such as that from WM region, a phenomenon which is commonly reported in ICA literature (Cole et al. 2010). Thus, in our study we chose an intermediary model order of 15 for identifying the major known large-scale networks while retaining the individual characteristics of SM brain.

Secondly this study uses anesthesia which is known to influence the measurement of functional connectivity (Hutchison et al. 2014). Recently, Hori et al. observed that in case of anesthesia, the global structure of a resting state network may be conserved, but some nodes of the network may be lost (Hori, Schaeffer, Gilbert, et al. 2020). Also, Giacometti et al. reported frontal cortical functional connectivity is impacted by anesthesia in macaques (Giacometti et al. 2021). In our study we use a light level of anesthesia in which BOLD functional responses are readily detectable although that may also result in some networks not being activated in whole. We have previously observed high correspondence between connectivity measures of rsfMRI signals and local field potentials obtained under similar anesthetic conditions (Wu et al. 2019). Moreover, there are certain challenges due to use of anesthesia. Previous studies (Belcher et al. 2013) which have used awake monkeys have reported that monkeys retain a very small degree of freedom of movement and even those small amounts of motion can produce artificial correlations in resting state time courses (Power et al. 2012). They also mentioned that shifting levels of arousal could have varying effects on ICA results in awake animals.

Thirdly, although the tSNR was generally high (mean value = 170 ± 68) after pre-processing (Supplementary Fig. S3) with no systematic difference between cortical, sub-cortical and WM regions, the brain periphery was found to have lower values than the rest of the brain. While this didn’t affect detecting any networks from the brain periphery such as the occipital pole (IC15) or orbito-frontal cortex (IC12) network, it may have resulted in their low connectivity with other networks. The low tSNR in these locations is most likely because of their distance from center of the coil which can be mitigated by improving the hardware configurations.

## Conclusion

The current study provides a detailed demonstration of resting state networks from squirrel monkey using ICA of whole brain fMRI data at millimeter resolution. We identified 15 reproducible networks covering the entire SM cortex as well as subcortical contributions from the thalamus, cerebellum, basal-ganglia structures and others with close correspondence to anatomical and functional landmarks. Importantly, these networks were found to compare well with networks obtained from human and other NHP models such as macaques and marmosets suggesting that SM brain maintains the same basic pattern of resting state fluctuations as observed in other species. Thus, this study adds to the information available from fMRI on the development of brain functional organization across species and confirms the value of SMs as a suitable animal species for evaluating a range of manipulations not possible in human subjects.

## Supplementary Material

Supplementary_Material_tgad018

## Data Availability

The data underlying this article will be shared on reasonable request to the corresponding author. Dr Anirban Sengupta.
