# Supplementary material for: Detection and characterization of resting state functional networks in squirrel monkey brain
Source: Cereb Cortex Commun. 2023 Sep 2;4(3):tgad018. doi: 10.1093/texcom/tgad018 (PMC10518810; doi:10.1093/texcom/tgad018)
Supplement: Supplementary_Material_tgad018 [file supplementary_material_tgad018.docx]

**Supplementary Material**

| **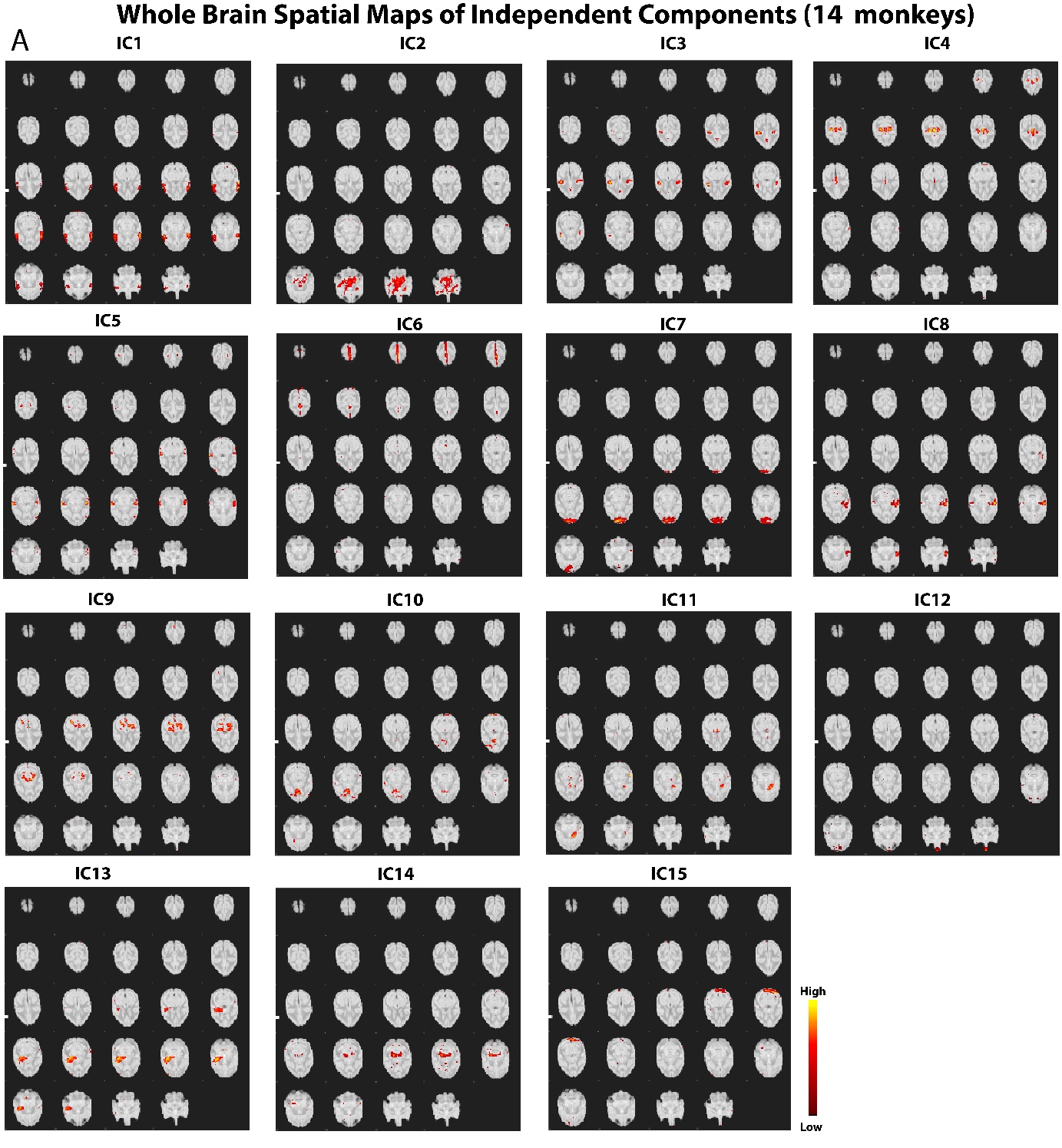** |
| --- |
| **Supplementary Figure S1: 15** Independent Components detected using ICA (N=14). (A) Spatial location of each of the 15 Independent Components from whole brain (24 slices) in Axial orientation (thresholded at Z=3). All the components are overlayed on squirrel monkey brain (VALIDATE) atlas (Schilling et al. 2017) for display purpose. |

**ICA with 14 and 16 animals – Robustness study**

We recruit naïve monkeys regularly in our lab. Initially 14 such naïve such monkeys were used to detect the GM and WM hubs/ROIs using ICA the results of which are shown in the main manuscript (**Figure 1A**) and in details in **Supplementary Figure S1**. Once the ROIs were detected, we performed connectivity analysis between the ROIs in those 14 animals as well as 2 more naïve monkeys that were acquired later, making it a total of 16. The rationale is once the ROIs gets standardized from a sufficient sample size of monkeys, these ROIs can be used for future connectivity analysis in other naive monkeys from the same species.

In-order to demonstrate our hypothesis regarding generalization of the detected ROIs, we performed ICA on all the 16 animals (43 runs) including the 2 new animals. What we found is a gross similarity between the ROIs obtained before using 14 animals (37 runs) and those obtained now using the 16 animals (43 runs) at the same threshold. The **Supplementary Figure S2** shows the 15 ROIs detected from 16 animals with the name of the original IC that they correspond to spatially. All the original 15 ICs (from 14 animals) are reflected in the new ROIs (from 16 animals) as individual separate components with only IC15 being clubbed with IC10 as a combined IC. Also, IC8 which was unilaterally expressed in the original ICA is expressed as 2 components (named IC8 R and IC8 L in Fig B) each located on one side of the brain. Thus, we found that the sample size of 14 monkeys was large enough to obtain the standardized ROIs, as adding new monkeys to the sample size didn’t alter the spatial location of the ICs with only mere difference in the IC decomposition process which is well known from ICA literature (Smith et al. 2009; Tian et al. 2013).

| 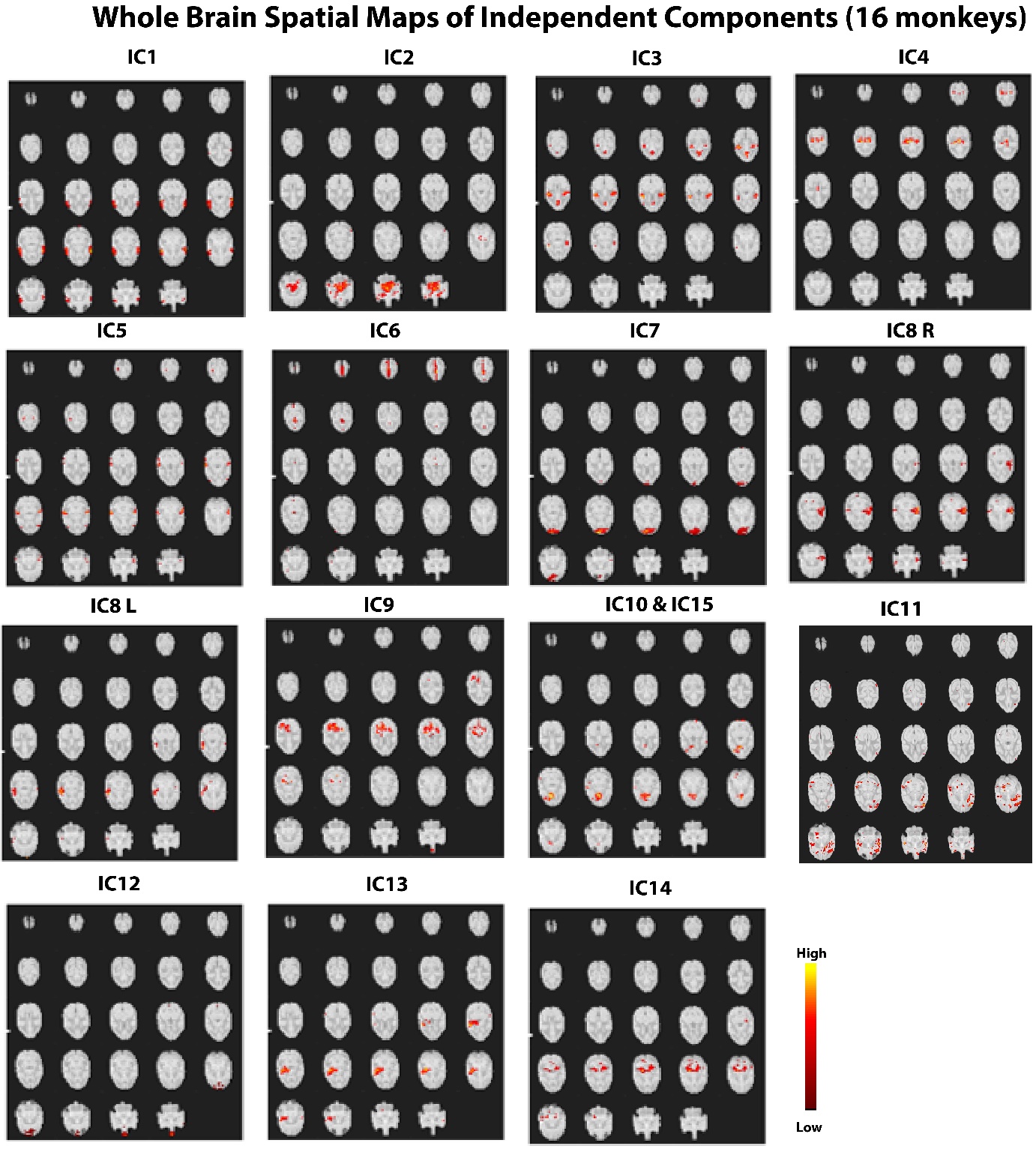 |
| --- |
| **Supplementary Figure S2:** Spatial location of each of the 15 Independent Components from whole brain (24 slices) of 16 animals (2 new animals added to the previous cohort of 14) thresholded at Z=3. The components are named based on the IC number that it corresponds to spatially from the initial analysis with 14 animals. |

**Temporal SNR (tSNR) analysis from different brain regions**

We studied the tSNR of brain images to investigate any pattern that may affect our results. Below is the tSNR from a representative central brain slice, with values from different regions provided. The tSNR is generally high with a mean value of 170+/-68 within the brain after pre-processing. There is no systematic difference between cortical, subcortical or white matter regions of the brain as shown in the table. However, the tSNR is generally lower in the brain peripheral regions at the anterior or posterior regions. We suspect this is more due to the hardware and coil limitations and their larger distance from the center of the coil. While this didn’t affect detecting any networks from the brain periphery such as the occipital pole (IC15) or orbito-frontal cortex (IC12) network, it may have resulted in their low connectivity with other networks.

| 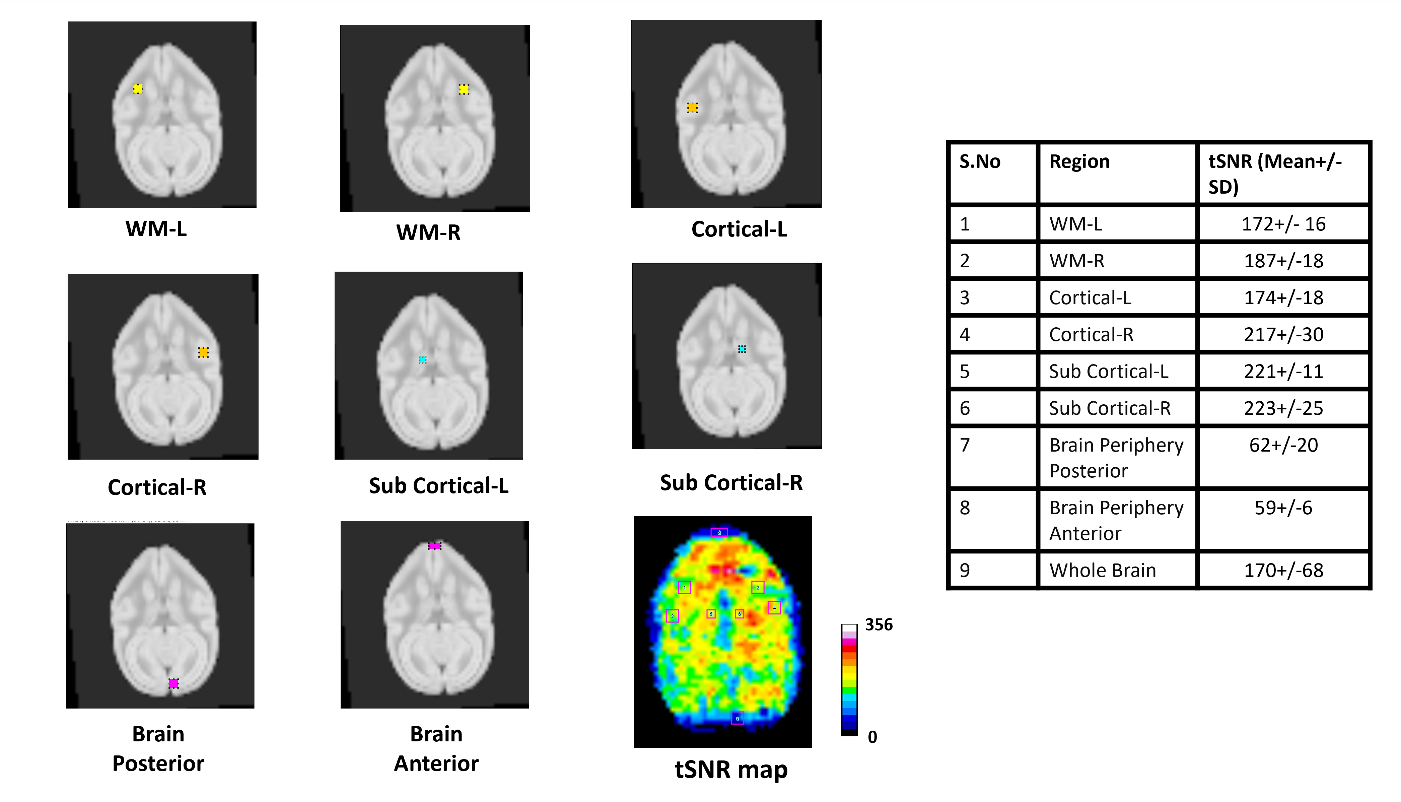 |
| --- |
| **Supplementary Figure S3:** Temporal SNR from different regions of a representative central brain slice. The drawn ROIs are shown overlayed on the anatomical images. Left is denoted as L and right is denoted as R. The tSNR map is color coded. The tSNR values from different regions are provided in the table. |

**ICA with a reduced model order**

We analyzed our data with a reduced ICA model order of 10 to see the differences from using a model order of 15 which we used in our study. We found that certain sub-networks get combined using the reduced model order. Thus, IC3 and IC6 from our study which are parts of somatosensory network appear together as a single component with a 10-model order as shown in Fig **Supplementary Figure S4 A**. Also, IC7 (vmPC) with some overlap with IC12 (front-orbital pole) appears as a single component in Fig **Supplementary Figure S4 B**, thus representing executive control network. However, IC12 is also expressed in overlap with IC15 (occipital pole) and with IC2, thus not appearing as a separate component (not shown) when we use a model order of 10. Moreover, WM components such as IC10 or subcortical components such as brainstem (IC14) are not detected as separate components with a model order of 10. Thus, while certain networks get expressed as combination of its sub-parts, a lower model order can also lead to less conspicuous networks staying undetected as we know from previous ICA literature (Cole et al. 2010; Tian et al. 2013).

| 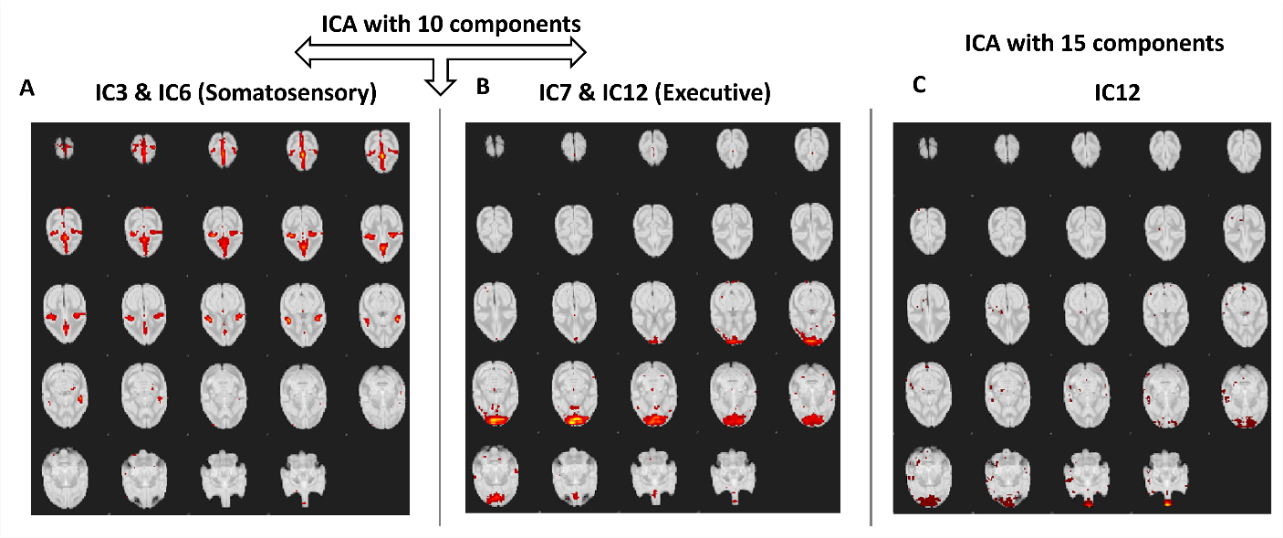 |
| --- |
| **Supplementary Figure S4: (**A) and (B) show two independent components detected after doing ICA with 10 components. (A) shows regions previously depicted in IC3 and IC6 while (B) shows IC7 with some overlap with IC12. Components are thresholded at Z=2. (C) is IC12 from the original study (ICA with 15 components) thresholded at a Z=2. |

It needs to be also mentioned that when we reduce the Z threshold to 2 (Z was 3 in manuscript) with the model order 15 (as used in our study), we see overlap of IC12 with IC7 in some slices of the brain a as shown in **Supplementary Figure S4 C**, thus reflecting the executive network. We also observe strong inter-network correlation (r=0.63) between IC4-IC6 which are subparts of the DMN with the model order 15. Thus, some of the bigger networks can also be inferred with a model order of 15 if we combine this information along with the component’s spatial locations.

**References**

Cole DM, Smith SM, Beckmann CF. 2010. Advances and pitfalls in the analysis and interpretation of resting-state FMRI data. Front Syst Neurosci. 4:8.

Schilling KG, Gao Y, Stepniewska I, Wu TL, Wang F, Landman BA, Gore JC, Chen LM, Anderson AW. 2017. The VALiDATe29 MRI Based Multi-Channel Atlas of the Squirrel Monkey Brain. Neuroinformatics. 15:321–331.

Smith SM, Fox PT, Miller KL, Glahn DC, Fox PM, Mackay CE, Filippini N, Watkins KE, Toro R, Laird AR, Beckmann CF. 2009. Correspondence of the brain’s functional architecture during activation and rest. Proc Natl Acad Sci U S A. 106:13040–13045.

Tian L, Kong Y, Ren J, Varoquaux G, Zang Y, Smith SM. 2013. Spatial vs. Temporal Features in ICA of Resting-State fMRI - A Quantitative and Qualitative Investigation in the Context of Response Inhibition. PLoS One. 8.
